# Project INTEGRATE - a common methodological approach to understand integrated health care in Europe

**DOI:** 10.5334/ijic.1980

**Published:** 2014-12-15

**Authors:** Lucinda Cash-Gibson, Magdalene Rosenmoller

**Affiliations:** IESE Business School, University of Navarra, Barcelona, Spain; IESE Business School, University of Navarra, Barcelona, Spain

**Keywords:** delivery of health care, integrated, integrated care, organisational case studies, Europe, health policy

## Abstract

**Background:**

The use of case studies in health services research has proven to be an excellent methodology for gaining in-depth understanding of the organisation and delivery of health care. This is particularly relevant when looking at the complexity of integrated healthcare programmes, where multifaceted interactions occur at the different levels of care and often without a clear link between the interventions (new and/or existing) and their impact on outcomes (in terms of patients health, both patient and professional satisfaction and cost-effectiveness). Still, integrated care is seen as a core strategy in the sustainability of health and care provision in most societies in Europe and beyond. More specifically, at present, there is neither clear evidence on transferable factors of integrated care success nor a method for determining how to establish these specific success factors. The drawback of case methodology in this case, however, is that the in-depth results or lessons generated are usually highly context-specific and thus brings the challenge of transferability of findings to other settings, as different health care systems and different indications are often not comparable. Project INTEGRATE, a European Commission-funded project, has been designed to overcome these problems; it looks into four chronic conditions in different European settings, under a common methodology framework (taking a mixed-methods approach) to try to overcome the issue of context specificity and limited transferability. The common methodological framework described in this paper seeks to bring together the different case study findings in a way that key lessons may be derived and transferred between countries, contexts and patient-groups, where integrated care is delivered in order to provide insight into generalisability and build on existing evidence in this field.

**Methodology:**

To compare the different integrated care experiences, a mixed-methods approach has been adopted with the creation of a common methodological framework (including data collection tools and case study template report) to be used by the case studies for their analyses.

**Methods of analysis:**

The four case studies attempt to compare health care services before and after the ‘integration’ of care, while triangulating the findings using quantitative and qualitative data, and provide an in-depth description of the organisation and delivery of care, and the impact on outcomes. The common framework aims to allow for the extraction of key transferable learning from the cases, taking into account context-dependency.

**Conclusion:**

The application and evaluation of the common methodological approach aim to distill and identify important elements for successful integrated care, in order to strengthen the evidence base for integrated care (by facilitating cross-context comparisons), increase the transferability of findings from highly context-specific to other settings and lead to concrete and practical policy and operational recommendations.

## Introduction

The concept of integrated care is widely used in different health systems in different ways, while a common universally accepted definition is absent. ‘*Project INTEGRATE-Benchmarking Integrated Care for better Management of Chronic and Age-related Conditions in Europe*’ [1] is a collaborative project under the European Commission Seventh Framework Programme (FP7) that aims to define what constitutes good quality integrated care provision, by gaining valuable insights into integrated care especially in terms of care process design, service delivery, professional skills mix, patient involvement, funding flows, regulatory conditions and enabling information communication technology. Further learning in these areas will help to create and improve connectivity, alignment and collaboration within and between the health and social care sectors, and thus bring benefits to patients, as well as to European health and social security systems faced with the challenges of an ageing population and an increase in chronic conditions.

To ensure a common understanding and improve the conceptualisation of the entire process, Project INTEGRATE uses Kodner's definition of integrated care: ‘*a coherent set of methods and models on the funding, administrative, organisational, service delivery and clinical levels designed to create connectivity, alignment and collaboration within and between the cure and care sectors*’ [2]. The Project is organised into three phases as follows:

*Phase 1*: A conceptual framework and common methodological framework is developed to support the development (and later cross-comparisons) of four different case study reports.

*Phase 2*: Building on the four case studies, Project INTEGRATE looks into range of ‘horizontal’/‘cross-cutting’ items, as they emerge. These items, considered by the literature as crucial for integrated care [3–5], form part of the main components of integrated health systems [6]: *process management, human resources management, financial flows*, *patients’ involvement and* *information communication technology management*.

*Phase 3*: Phase 1 and 2 findings are contrasted with international evidence and feed into operational and policy recommendations to support the development, adoption and successful management of integrated care in other settings.

**Figure 1.**
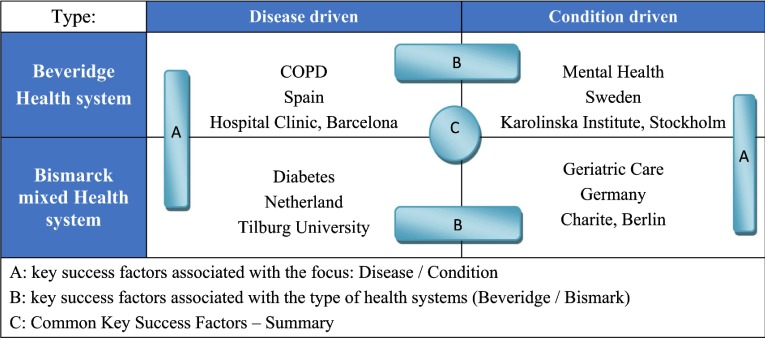
Classification of the Project INTEGRATE case studies and comparisons to be made. (Source: Project INTEGRATE documentation)

Project INTEGRATE looks into four different integrated care settings across four countries, which include two disease-pathways and two general care co-ordination conditions. The selected disease and care co-ordination concepts address several conditions - chronic obstructive pulmonary disease (COPD), diabetes, geriatric condition and mental care - that are among those with a high epidemiologic importance and high economic burden on health systems [7, 8]. Regarding country selection, two different types of national health systems (Spain and Sweden) and health insurance systems (the Netherlands and Germany) are being considered. The rationale for these choices are that these experiences are likely to demonstrate different models for integrated care success and thorough comparing and contrasting project findings, reveal common key success factors as well as the ‘context’-specific success factors (Figure 1 for a visual representation of the different elements Project INTEGRATE aims to compare).

**Figure 2.**
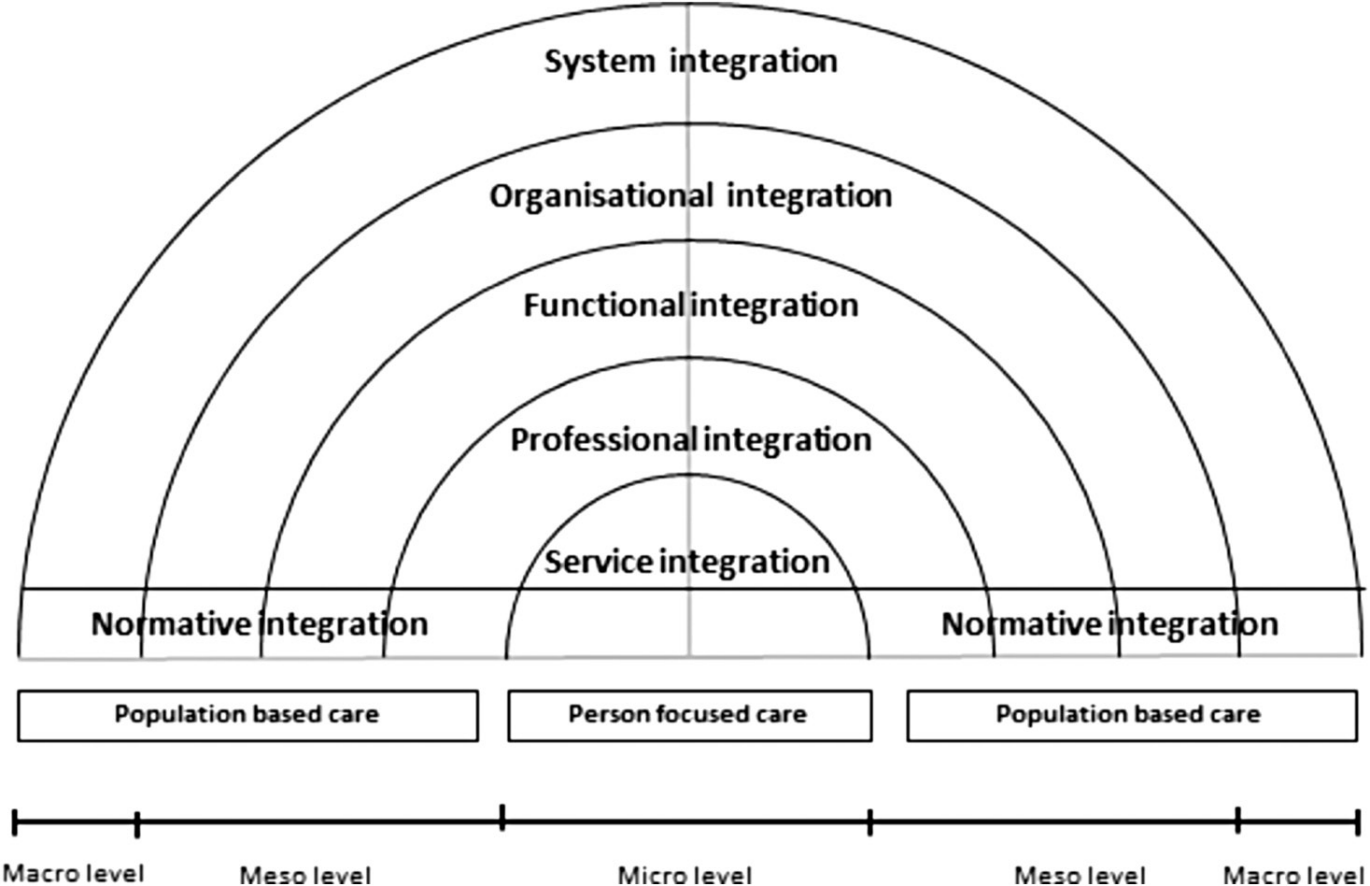
The levels of integrated care-concepts for data analysis (adapted from Valentijn [9])

Based on the comparison of the four case study findings, Project INTEGRATE seeks to gain further knowledge of the different levels of integrated care/integration, through analysis of the corresponding horizontal cross-cutting themes (Figure 2).

- Personal integration/person-centred care (i.e. bio-psycho-social integration of needs of patients) - within Phase 2 ‘patient involvement’ is examined;
- Service integration (i.e. provider care that is integrated into a coherent process) - within Phase 2 ‘Care process design’ is examined;
- Professional integration (i.e. multi-professional teams/networks with the right skill-mix) - within Phase 2 ‘Human resources/workforce management and development’ is examined;
- Functional integration (i.e. non-clinical support and back office functions to support integrated care) - within Phase 2 information communication technology management is evaluated;
- Organisational integration (i.e. how and where organisations/providers are brought together) - within Phase 2 financial flows and payment systems is analysed; and
- System integration (i.e. coherence of health policies from different levels) - lastly within Phase 3 Project INTEGRATE forms policy and operational recommendations.

The intention is to operationalise this process by identifying relevant items for integrated care at each of the different levels and sources and to examine the interplay between different levels over time and in the different contexts.

This paper describes the common approach (methodology, accompanying instruments and template report) used by Project INTEGRATE case studies, to ensure coherent and consistent data collection, in order to obtain high quality results that can then be compared and contrasted with international evidence and fed into operational and policy recommendations.

## Methodology and methods of analysis

Integrated care is multifaceted and thus its analysis requires insight and understanding from multiple perspectives. The use of case study methodology in combination with a mixed-methods approach is one way to first generate these essential multiple perspectives, second it allows for triangulation of the perspectives - whilst overcoming some of the specificity derived from diverse case studies - so that they may be mutually corroborated and led to appropriate use and transfer to other settings. The successful use of this approach in Project INTEGRATE also aims to encourage and facilitate further (necessary) cross-context comparisons in complex health system research (such as in integrated care research) to gain new in-depth understandings of the organisation and delivery of health care.

### Case study (report) development

Project INTEGRATE has established the following minimum required information to be collected, undertaken by a common approach to facilitate the collection of similar (and thus comparable) data from each case study. A detailed case study report template has also been developed (Annex 1: Detailed case study report template).

- Working definition of integrated care: The specific case study context, the related objectives of the programmes or target group;
- Description of the implementation of the integrated care case: This includes a detailed understanding of: (i) the reasons and influences behind the choice of integrated care; (ii) why and how this particular approach was developed and implemented (implementation strategies); and (iii) how care services, professional roles, organisational arrangements and support systems are redesigned. This includes a time diagram to indicate the previous, current and desired status, where key elements and differences are shown to allow broad level comparison;
- Context influences which helped and hinder implementation;
- Describe the care intervention, organisation and management of integrated care.
- Assess the impact on professionals, and other key stakeholders, patient and professional experiences and care outcomes, and cost-effectiveness. Assess the impact in terms of value added and costs incurred, and listing of other possible causes of these impacts, other than the integration changes.
- Identify key barriers and key facilitators to the effective development of integrated care, and how these were overcome or were respectively encouraged.

### Mixed-methods approach: data collection tools for case studies

As part of the common methodology framework and mixed-methods approach, project partners prospectively agreed that it would be beneficial to create a common strategy for the following methods.

### Literature review strategy

The four case study reports are accompanied by a literature review to gain an overall in-depth analysis of the case studies and build on the existing body of knowledge in the respective fields of each case condition, taking into account existing valuable approaches [10]. The literature review approach uses an operational version of the definition of integrated care and is intended to be adapted by each case study site as appropriate (Annex 2: for details of the Common Literature Review Strategy).

Project partners acknowledge that there are some limitations to the common search strategy; the decision to link the definition of integrated care to the Chronic Care Model [11] might limit the researchers to certain aspects of integrated care that are not described by the model (although the model was chosen based on its international scientific acceptance and relevance). The limitation to two core components of the Chronic Care Model [11] has also been discussed: on one side, it might limit the scope of study, while on the other it helps focus the analysis. It has been concluded that the search is likely to still identify programmes with the other elements of the Chronic Care Model (of the health system and community components) [11], even if they are not explicitly stated in the search strategy.

### Retrospective (chronic care) process and data analysis

Project INTEGRATE aims to acquire illustrative information about the pre/post integrated care intervention; this includes any available process and outcome data and administrative data (such as cost), through the utilisation of clinical databases and information system (Annex 1: for additional guidance on this).

### Semi-structured interviews with stakeholders

Semi-structured interviews aim to gain insights into each stakeholders’ understanding of and role in integrated care across past, current and future settings. Interviews had as main topics the access to individual care services; the situation before implementing integrated care, details about the implementation, any relevant context, the care co-ordination, patient involvement, the information communication technology in place, the financial flows, and the facilitators and barriers to successful and suitable integrated care. Interviews are foreseen with patients, health care professionals managerial and other staff who were involved in the co-ordination and delivery of integrated care services. For instance in the COPD/Spain case study, 10 interviews are foreseen with: two patients, one case manager nurse/head of integrate care unit, two integrated care unit nurses, one integrated care unit physician, one respiratory medicine specialist, one primary care nurse, one primary care case manager and one primary care physician. This will not be the same/relevant for the other case studies. Common interview question templates, interviewee information sheets and consent forms have been created to support the interview process.

## Conclusion

In Project INTEGRATE a ‘common’ mixed-methods approach is being used – a conceptual framework, common methodology framework and common case study report template – by the four different case studies as a way of overcoming the usual challenge of context specificity and limited transferability of findings to other settings. Case study methodology combined with a mixed-methods approach can be extremely useful and relevant for complex health services research (i.e. research on integrated care), as it starts to provide an in-depth and broad understanding of the organization and delivery of care and helps to untangle some of the web of multifaceted complexity associated with integrated health care programmes. This is urgently needed, since these types of programmes are currently being widely implemented at different levels of ‘maturation’ (and understanding) in different health services worldwide [12]. Thus the approach described in this paper can be used as a way to ultimately derive key lessons and markers for successful application of integrated care programmes (including insight into barriers and facilitators), for different delivery contexts, in different types of health systems and for different patient-groups.

## Annex 2. Common literature review strategy

Within the common literature review(s) strategy the following guiding questions are used:

- Which forms of integrated care for [COPD/diabetes/mental health/geriatric care] have been identified in the literature?
- What are the interventions/implementation strategies that are described in integrated care programmes for [COPD/diabetes/mental health/geriatric care] respectively?
- What are the barriers and facilitators to integrated care that are described in integrated care programmes for [COPD/diabetes/mental health/geriatric care] respectively?
- What are the outcomes of the interventions/implementation strategies that are described in integrated care programmes for [COPD/diabetes/mental health/geriatric care] respectively?

We adopted a systematic approach to identify the references to be included, where appropriate, and the search was not limited by language. The following sources were considered: peer-reviewed published articles, studies, publications, etc. and reference reviews elaborated by scientific leaders in the field (e.g. The Kings Fund (UK), The Nuffield Trust (UK), Nivel (NL).

The common search strategy includes three main sets of terms:

- ***Health condition:*** Depending on the case study condition.
- ***Components of the Chronic Care Model*** [11, 14]: including at least two core components:
  - *Self-management*: self-management, self-care, self-management, self-management support, patient-centeredness, patient-centred care, patient involvement, patient education, information provision, behavioural support, behaviour modification, health literacy, cultural sensibility, stress management, motivational support.
  - *Delivery systems design*: delivery system design, care pathway, critical pathway, individualised care plan, access to care, care co-ordination, clinical case management services, medicines management, case finding, co-morbidities management, regular follow-up, planned interactions, patient care planning, practice team, patient care team, team roles, team change, professional roles, practice nurse counselling, team-based care provision.
  - *Decision support*: decision support, evidence-based guidelines, guideline adherence, standards, patient preference, clinician reminders, patient reminders, reminder systems, provider education, feedback, speciality expertise integration, referral, barriers to care, performance review, individualised care plans, patient participation.
  - *Clinical information systems*: clinical information system, clinical registry, registries, population information database, patient data, population data, information sharing, shared information system, health information systems, health information technology electronic registry, clinical reminder, patient reminder, clinician reminder, provider feedback, performance monitoring, monitoring system, information communication technology devices, patient portal, telemonitoring, telehealth, teleassistance, telehomecare, videoconferencing, mobile phone, patient-held record.
- ***Type of intervention***: Integrated care is frequently used as an umbrella term; therefore within the search strategy the following terms will be considered: Integrated Care, Care co-ordination, Disease management, Disease state management, Comprehensive healthcare, Complex interventions, Multifactorial lifestyle interventions, Shared Care, Chronic Care Model, Care transition, Transitional care, Intermediate care, Case management.
